# Response to: Metacognition in functional cognitive disorder: contradictory or convergent experimental results?

**DOI:** 10.1093/braincomms/fcac139

**Published:** 2022-05-25

**Authors:** Rohan Bhome, Andrew McWilliams, Gary Price, Norman A. Poole, Robert J. Howard, Stephen M. Fleming, Jonathan D. Huntley

**Affiliations:** Dementia Research Centre, University College London, 8-11 Queen Square, London, UK; Wellcome Centre for Human Neuroimaging, University College London, London, UK; Department of Psychological Medicine, Institute of Psychiatry, Psychology and Neuroscience, King’s College London, London, UK; UCL Institute of Child Health, Great Ormond Street, London, UK; Department of Experimental Psychology, University College London, London, UK; National Hospital for Neurology and Neurosurgery, London, UK; South West London and St George's Mental Health NHS Trust, London, UK; Division of Psychiatry, University College London, London, UK; Wellcome Centre for Human Neuroimaging, University College London, London, UK; Department of Experimental Psychology, University College London, London, UK; Max Planck University College London Centre for Computational Psychiatry and Ageing Research, London, UK; Division of Psychiatry, University College London, London, UK

Dr Larner^1^ highlights the findings of a recent paper by Pennington *et al*.^2^ in which the authors ‘did not find metacognitive deficits in groups of well-characterized patients with functional cognitive disorder (FCD)’ and suggests that this is both contradictory to our findings and places our proposed Bayesian account in jeopardy.^3^ In fact (as Dr Larner later admits), the findings from the two studies are convergent. In both our^3^ and Pennington *et al*.’s^2^ studies, local metacognition—the extent to which trial by trial ratings of confidence covary with task performance—was measured in people with FCD. In both studies, across both perceptual and memory tasks, local metacognitive efficiency (meta-d’/d’) was unimpaired relative to controls. As Dr Larner points out, it is difficult to place too much reliability on null results with small samples, which might reflect a consequence of Type 2 errors. Replication of intact local metacognition in FCD across two distinct samples is therefore noteworthy, and we were pleased to see Pennington *et al*.’s data.^2^

As Dr Larner further highlights, we found that people with FCD had deficits in global metacognition, which was not investigated by Pennington *et al*.^2^ The reasons for this dissociation remain unclear, and understanding this linkage will benefit from novel tasks that allow the relationship between local and global metacognition to be quantified.^4,5^ However, the Bayesian model we proposed in our paper sought to accommodate the observed null findings with respect to local metacognition—and is therefore supported, rather than contradicted, by Pennington *et al*.’s convergent findings.^2^ There are no doubt alternative possibilities, and the suggestion that neural networks might overfit to experience due to impaired sleep and dreaming, leading to selective impairment in global metacognition, is certainly interesting and warrants further investigation.

## Data availability

Data sharing is not applicable to this article as no new data were created or analysed.
